# Safety profile of vascular endothelial growth factor receptor tyrosine-kinase inhibitors in pediatrics: a pharmacovigilance disproportionality analysis

**DOI:** 10.3389/fphar.2023.1160117

**Published:** 2023-06-12

**Authors:** Yifei Xue, Shuo Feng, Guangyao Li, Chao Zhang

**Affiliations:** Department of Pharmacy, Beijing Tongren Hospital Affiliated to Capital Medical University, Beijing, China

**Keywords:** pharmacovigilance, FDA adverse event reporting system (FAERS), VEGFR tyrosine kinase inhibitors, pediatrics, adverse event (AE)

## Abstract

**Introduction:** existing research on children consists primarily of phase I/II clinical trials for VEGFR-TKI. System reports of safety on the use of VEGFR-TKI in pediatrics are lacking.

**Aim:** to investigate the safety profiles of VEGFR-TKI in pediatrics via the FDA Adverse Event Reporting System (FAERS).

**Method:** data regarding VEGFR-TKIs were extracted from the FAERS between 2004Q1 to 2022Q3 and categorized by the Medical Dictionary for Regulatory Activities (MedDRA). Population characteristics were analyzed, and reporting odds ratio (ROR) was performed to identify risk signals associated with VEGFR-TKI.

**Results:** 53,921 cases containing 561 children were identified in the database from 18 May 2005, to 30 September 2022. Among those in the system organ class, skin, subcutaneous tissue disorders, and blood and lymphatic system disorders in pediatrics contributed to over 140 cases. Palmar-plantar eythrodysesthesia syndrome (PPES) in VEGFR-TKI presented the most significant 340.9 (95% 229.2–507.0). And pneumothorax also gave a high reporting odds ratio of 48.9 (95% 34.7–68.9). For a specific drug, musculoskeletal pain gave a ROR of 78.5 (95% 24.4–252.6) in cabozantinib and oesophagitis in lenvatinib with a ROR of 95.2 (95% 29.5–306.9). Additionally, hypothyroidism presented a high signal, especially sunitinib, with a ROR of 107.8 (95% 37.6–308.7).

**Conclusion:** the present study explored the safety profile of VEGFR-TKI in pediatrics using the FAERS database. Multiple skin and subcutaneous tissue disorders, as well as blood and lymphatic system disorders, were common VEGFR-TKI-related AEs in system organ class. No serious hepatobiliary AEs were detected. For the specific AEs, PPES and pneumothorax were VEGFR-TKI-related AEs that presented significantly higher signals than those in the general population.

## 1 Introduction

Vascular endothelial growth factor (VEGF) and its receptor have crucial roles in the growth and subsequent physiologic homeostasis in endothelial cell neogenesis, angiogenesis, and neovascularization, as well as pathologic processes, such as cancer and ophthalmic disorders (Folkman, 1972; Ferrara, 2004). Widespread use of these agents has improved survival rates and is well-tolerated by a range of advanced adult cancers (Ferrara and Adamis, 2016). The reported efficacy of pazopanib, lenvatinib, and anlotinib also supported clinical use in children with solid tumors (Weiss et al., 2020; Gaspar et al., 2021a; Xu et al., 2021). Multiple meta-analyses have demonstrated that multi-target VEGFR-TKI in adults was linked to hand-foot skin reactions (HFSR), rashes, bleeding, hypertension, and cardiotoxicity in adults (Massey et al., 2015; Das et al., 2021; Hou et al., 2021). However, given the low prevalence and heterogeneity of pediatric cancers (Spini et al., 2022), there has not yet been enough research on the safety profile of anti-angiogenesis therapy in pediatric patients.

Pharmacovigilance is defined as the science and activities relating to the detection, assessment, understanding, and prevention of adverse effects or any other drug-related problem (Bohm et al., 2021). Pharmacovigilance is an essential component of drug safety monitoring, providing early identification of potential drug-related adverse events (AE) through active and voluntary surveillance efforts in a real-world setting (Dhodapkar et al., 2022). The FDA adverse event reporting system (FAERS) is a well-known AE spontaneous reporting system that documents numerous drug AE reports and medication errors and contains the largest and most normative data (Sakaeda et al., 2013). Disproportionality analysis in data mining algorithms (DMAs) such as proportionality reporting ratio (PRR) with associated χ^2^ value and reporting odds ratio (ROR) with 95% confidence interval (CI) are used to generate a hypothesis for risk signals using FAERS database (Sharma et al., 2023). We sought to explore the safety of VEGFR-TKI via FAERS while focusing on potential AEs associated with using these drugs in pediatric patients.

The objective of this real-world pharmacovigilance for children aimed to systemically investigate the association of reported AEs and FDA-approved multi-target VEGFR-TKI based on the FAERS database. Clarifying potential AEs associated with VEGFR-TKI of the study can provide evidence for future clinical research and enable clinicians to select the most effective therapies in clinical practice.

## 2 Methods

### 2.1 Date source

Seven VEGFR-TKI, including sunitinib, sorafenib, pazopanib, regorafenib, axitinib, cabozantinib, and lenvatinib, have partly similar targets to anlotinib, were selected as study drugs. In the FAERS database, drug names are arbitrary, and both generic and brand names are included as the keyword in the subsequent analysis. In OpenVigil, drugs are named according to the U.S. Adopted Name (USAN) scheme. Hence, we directly leveraged OpenVigil to rely on the FAERS database for mapping the drug names to USAN. Finally, OpenVigil 2.1-MedDRA-v24 was implemented, comprising 258,346 children (age 1–17) in 5,236 256 cases with case_id deduplicated of FAERS data from 2004Q1 to 2022Q3. Because it is impossible to identify individual patients, informed consent was not required.

### 2.2 Data cleaning of FAERS

The data cleaning and mapping of drug names in FAERS were done by OpenVigil 2.1 (Bohm et al., 2016; Bohm et al., 2021). This step filtered all duplicate and ambiguous reports that contain misspelled names of drugs and pharma products that are not corrected according to Drugbank or Drug@FDA. The AEs related to disease progression, tumor recurrence, off-label use, and other product use errors were excluded from the analysis. In addition, the Medical Dictionary for Regulatory Activities (MedDRA [v25.0]) was utilized to classify the AEs automatically into the broadest system organ class (SOC) and specific preferred term (PT) categories. In the FAERS database, PT is well-accepted and utilized, and both PT and SOC will be adopted to identify any possible AE.

### 2.3 Statistical analysis

Data mining to measure the association of VEGFR-TKI with specific AEs in the PT category was performed by disproportionality analysis. The reporting odds ratio (ROR) based on the 2 × 2 crosstab (Supplementary Table S1) was utilized to identify the signals which indicate a potentially increased risk of drug-associated AEs. And risk-signal detection ratio (RSR) was defined as the ratio of a risk signal to all PT reports in each drug. The computation of ROR and the criteria of a significant signal are shown as follows:

$$ROR=\frac{\left(DE∕dE\right)}{\left(De∕de\right)}\times 100\%$$

$$SE\left(\ln ROR\right)=\sqrt{\frac{1}{DE}+\frac{1}{De}+\frac{1}{dE}+\frac{1}{de}}$$

$$95\%CI=e^{\ln\left(ROR\right)\pm 1.96\times SE}$$

In population-specific outcomes, DE is the number of interest drug reports for suspect AE; dE is the number of other drug reports for suspect AE; De is the number of interest drug reports for other AE; de is the number of other drugs for other AE. It was considered significant if a potential risk signal simultaneously had ≥3 report cases, a ROR ≥2, a lower 95% confidence interval (CI) limit ≥1, and a Chi-square test (χ^2^) ≥ 4. Any death or pharmacodynamic-related PTs, such as disease and tumor progression, were excluded from the result of detection presentation.

### 2.4 Subgroup analysis

In the subgroup analysis, the risk signal results of patients in each age group would be counted according to the SOC level. Then the results of each age group were arranged according to the number of cases, and the top 10 AEs for VEGFR-TKI comprehensive results in each age group were compared. Due to the small number of cases of young patients in the FAERS database at this stage, patients aged 1–4 and those aged 5–11 were combined for discussion, and subgroup analysis was performed with the group aged 12–17.

## 3 Results

### 3.1 Data population characteristics for VEGFR-TKI

The characteristics of patients and AEs are presented in Table 1. We retrieved 53,921 cases involving 193,477 AE reports between 18 May 2005 and 30 September 2022. Moreover, 561 cases (1.04%, 561 of 53,921) involved pediatric patients, in which females made a total of 246 cases (43.85%), and 40 of 561 (7.13%) were unknown. Hospitalization in outcome analysis was most frequent in 154 cases (27.45%). Sorafenib was the drug that generated the most reports among the 7 VEGFR-TKI (37.79%, 212 of 561), followed by pazopanib (25.85%, 145 of 561). At least 33 countries and regions were represented in the FAERS data, with North America contributing the majority of reports (62.75%, 352 of 561), followed by Europe (22.99%, 129 of 561). Additionally, the indication used for osteosarcoma took most cases (15.15%, 85 of 561), followed by acute myeloid leukemia (8.20%, 46 of 561).

**TABLE 1:** Clinical characteristics of pediatric patients using VEGFR-TKI.

Characteristics	Case number (n)	Case proportion (%)
**All cases**	53,921	
**Pediatric cases**	561	
**Gender**		
Male	275	49.02
Female	246	43.85
Unknown	40	7.13
**Age**		
1–4	34	6.06
5–11	160	28.52
12–17	367	65.42
**Regions**		
Asia	59	10.52
Europe	129	22.99
North America	352	62.75
Oceania	7	1.25
South America	3	0.53
Unspecified	11	1.96
**Drugs**		
Sorafenib	212	37.79
Pazopanib	145	25.85
Cabozantinib	82	14.62
Lenvatinib	59	10.52
Sunitinib	27	4.81
Regorafenib	25	4.46
Axitinib	11	1.96
**Outcomes**		
Hospitalization	154	27.45
Death	114	20.32
Life-Threatening	19	3.39
Disability	4	0.71

### 3.2 Disproportionality analysis characteristics for VEGFR-TKI

**FIGURE 1:**
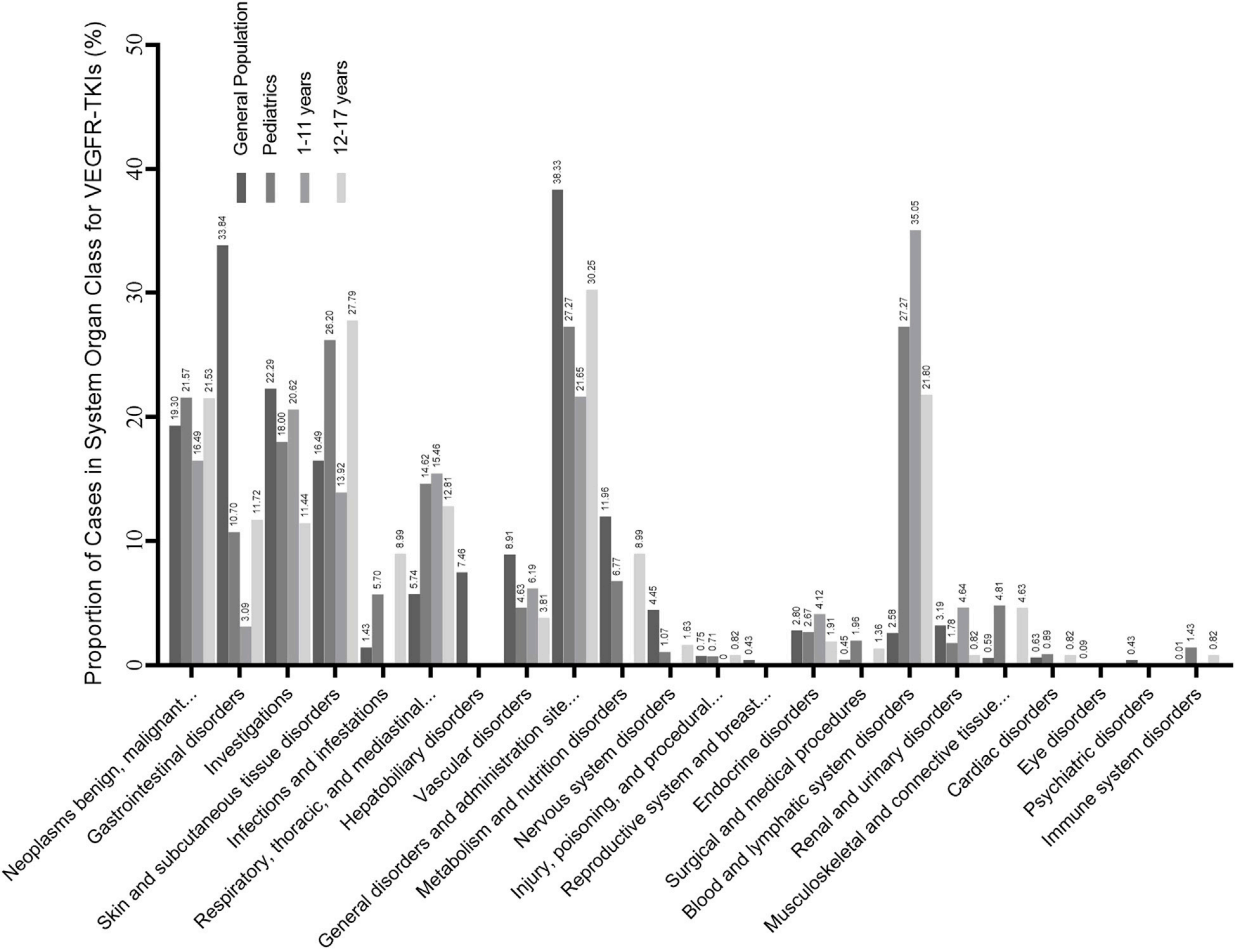
Proportion of cases in the risk preferred terms (PTs) identified in system organ class (SOC) level. The result is the ratio of the number of cases involved in the risk signal to the total number of cases. The general population contains all the records exported from the FAERS database. Subgroup analysis is also contained, and pediatrics is divided into the younger age group (1–11 years) and the older age group (12–17 years).

We detected AE signals for seven VEGFR-TKI in pediatrics. Figure 1 shows how the risk signals were distributed according to the SOC, and the quantity of cases is shown in Supplementary Table S3.

Disproportionality analysis results detected 5,276 PTs, and the pediatrics caught a total of 561 PTs, in which 99 potential risk signals (PTs) targeted 17 organ systems sorted by MedDRA. In pediatrics, 283 of 561 (50.45%) were injury, poisoning, and procedural complications (Supplementary Table S3), which took most cases at the SOC level, but most at the PT level were associated with off-label use. Compared to the results of the general population (3.21%, 1,389 of 53,921), the proportion of blood and lymphatic system disorders (27.27%, 153 of 561) was significantly higher.

**FIGURE 2:**
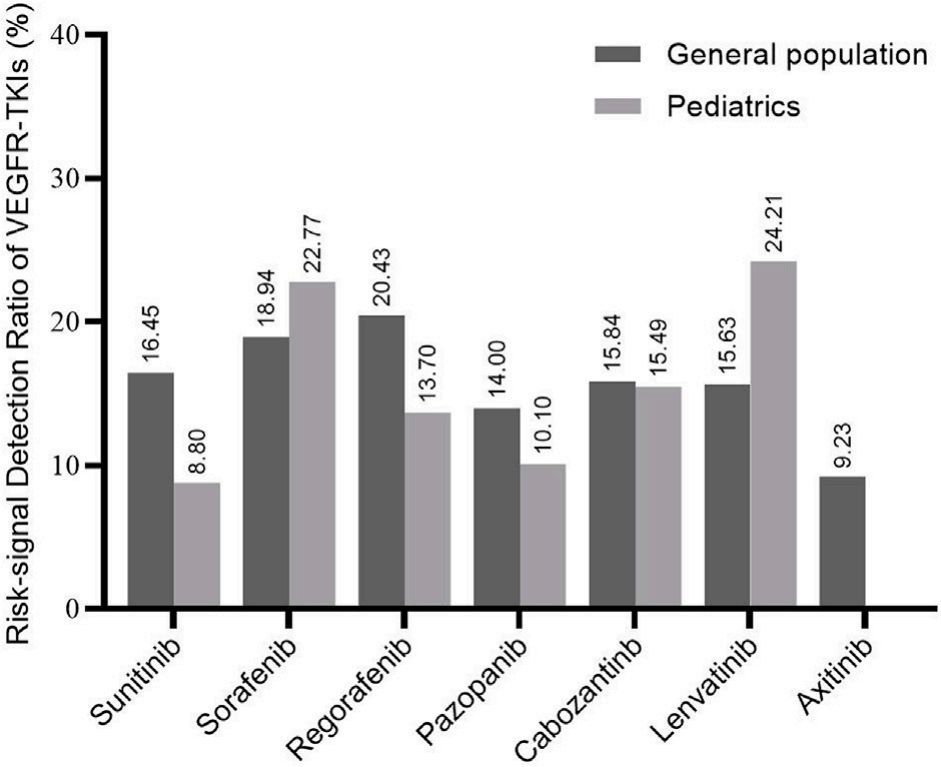
Risk-signal detection ratio for each VEGFR-TKI. The result is the ratio of the number of risk signals involved in each kind of VEGFR-TKI to the total recorded signals. The general population contains all the records exported from the FAERS database.

Figure 2 represents the RSR for each drug in the general population and pediatrics. The RSR for regorafenib (20.43%) was the highest among all included VEGFR-TKI in the general population. Axitinib detected the fewest risk PTs (9.23%) among the VEGFR-TKI. For pediatric patients, lenvatinib had the highest RSR detected (24.21%), followed by sorafenib (22.77%). Except for no significant difference in sorafenib, regorafenib, pazopanib, and cabozantinib between all populations and children, sunitinib in children were significantly lower than in the general population, whereas sorafenib and lenvatinib results were significantly higher (Supplementary Table S4).

### 3.3 Risk signal distribution in pediatric patients

We additionally presented the top 50 from a total of 99 risk signals in Table 2 for pediatrics and the comprehensive results of VEGFR-TKI based on adverse event reports. Skin and subcutaneous tissue disorders (n = 147) and blood and lymphatic system disorders (n = 153) were these steps’ two most prevalent SOCs. None of the risk signals has been identified in the result of axitinib.

**TABLE 2:** The ROR of the top 50 AE reports in pediatrics.

SOC	PT	VEGFR-TKI		Sunitinib		Sorafenib		Pazopanib			Cabozantinib		Regorafenib		Lenvatinib	
		N	ROR (95% CI)	N	ROR (95% CI)	N	ROR (95% CI)	N	ROR (95% CI)	N		ROR (95% CI)	N	ROR (95% CI)	N	ROR (95% CI)
**Investigations**	ALT increased	17	5.0 (3.1, 8.1)	1	5.1 ✗ (0.7, 37.3)	7	5.4 (2.5, 11.4)	2	2.2 ✗ (0.5, 8.8)	6		12.3 (5.4, 28.4)	1	6.6 ✗ (0.9, 48.7)		
	AST increased	11	3.5 (1.9, 6.4)	1	5.7 ✗ (0.8, 41.5)	3	2.5 ✗ (0.8, 7.8)			6		13.7 (6.0, 31.5)	1	7.3 ✗ (1.0, 54.1)		
	weight decreased	11	2.3 (1.3, 4.2)	3	11.9 (3.6, 38.9)	4	2.2 ✗ (0.8, 5.9)	3	2.4 ✗ (0.8, 7.5)	1		1.4 ✗ (0.2, 10.0)				
	platelet count decreased	10	4.3 (2.3, 8.0)	1	7.6 ✗ (1.0, 55.7)	2	2.2 ✗ (0.6, 9.0)	2	3.2 ✗ (0.8, 13.1)	1		2.9 ✗ (0.4, 20.6)			4	16.6 (6.0, 45.7)
	blood pressure increased	8	5.6 (2.8, 11.3)	2	25.7 ✗ (6.1, 107.9)	1	2.6 ✗ (0.4, 13.0)	1	2.6 ✗ (0.4, 18.9)	2		9.5 ✗ (2.3, 38.8)	2	33.6 ✗ (7.9, 142.7)		
	LDH increased	6	7.4 (3.3, 16.6)			4	13.1 (4.8, 35.3)	1	4.6 ✗ (0.6, 33.2)				1	28.2 ✗ (3.8, 209.2)		
	TSH increased	6	23.5 (10.3, 53.5)							5		138.8 (55.3, 348.1)	1	87.6 ✗ (11.8, 652.2)		
	neutrophil count decreased	6	4.7 (2.1, 10.5)			3	6.2 (2.0, 19.3)								3	22.3 (7.0, 71.3)
	blood bilirubin increased	5	4.1 (1.7, 9.9)			4	8.7 (3.2, 23.4)			1		5.5 ✗ (0.8, 39.5)				
	ejection fraction decreased	5	18.6 (7.6, 45.6)			3	29.2 (9.2, 92.3)	2	28.0 ✗ (6.9, 114.1)							
	hepatic enzyme increased	5	2.8 (1.2, 6.8)			4	6.0 (2.2, 16.1)	1	2.1 ✗ (0.3, 15.3)							
**Blood and Lymphatic**	anaemia	21	6.9 (4.4, 10.6)			10	8.6 (4.6, 16.3)	3	3.6 ✗ (1.2, 11.4)	1		2.1 ✗ (0.3, 15.3)	2	15.2 ✗ (3.6, 64.4)	5	15.6 (6.2, 39.0)
	bone marrow failure	23	12.4 (11.1, 25.8)	1	12.4 ✗ (1.7, 90.9)	18	36.2 (22.2, 59.0)	1	2.6 ✗ (0.4, 18.8)						3	19.9 (6.2, 63.7)
	febrile neutropenia	20	5.2 (3.3, 8.1)			10	6.9 (3.6, 13.0)	1	1.0 ✗ (0.1, 6.8)						9	24.2 (11.9, 49.2)
	neutropenia	18	3.9 (2.5, 6.3)	1	3.8 ✗ (0.5, 27.9)	12	7.1 (4.0, 12.7)	1	0.8 ✗ (0.1, 5.8)	1		1.4 ✗ (0.2, 10.4)			4	8.3 (3.0, 22.9)
	pancytopenia	21	11.2 (7.2, 17.4)	1	9.1 ✗ (1.2, 66.8)	20	29.8 (18.7, 47.4)									
	thrombocytopenia	16	4.6 (2.8, 7.6)	3	16.2 (4.9, 53.2)	6	4.5 (2.0, 10.2)	1	1.1 ✗ (0.2, 7.7)	4		7.9 (2.9, 21.7)	2	13.6 ✗ (3.2, 57.8)	2	5.3 ✗ (1.3, 21.7)
	lymphadenopathy	14	8.6 (5.0, 14.6)			13	21.8 (12.4, 38.3)	1	2.3 ✗ (0.3, 16.2)							
	lymphopenia	7	11.6 (5.5, 24.6)			6	26.6 (11.7, 60.2)						1	37.6 ✗ (5.1, 278.8)		
	myelosuppression	9	9.1 (4.7, 17.7)			5	16.5 (6.8, 40.4)	2	9.4 ✗ (2.3, 38.2)						1	11.4 ✗ (1.6, 82.3)
**Skin**	PPES	41	340.9 (229.2, 507.0)	2	183.5 ✗ (43.3, 777.4)	34	745.4 (484.4, 1,146.9)	1	18.7 ✗ (2.6, 134.6)	2		67.9 ✗ (16.5, 279.9)	2	239.3 ✗ (55.7, 1,027.7)		
	rash	29	2.3 (1.6, 3.3)			22	5.4 (3.5, 8.4)			5		3.0 (1.2, 7.4)	2	4.1 ✗ (1.0, 17.2)		
	alopecia	22	10.9 (7.1, 16.8)	2	17.6 ✗ (4.2, 73.6)	11	14.5 (7.9, 26.6)	8	15.2 (7.5, 31.2)				1	11.0 ✗ (1.5, 81.1)		
	skin toxicity	15	128.2 (72.5, 226.5)			14	323.5 (178.5, 586.5)			1		46.6 ✗ (6.4, 339.3)				
	hair colour changes	11	143.4 (73.3, 280.5)	1	186.3 ✗ (25.0, 1,390.6)	2	55.8 ✗ (13.5, 230.8)	5	220.6 (86.5, 562.7)	3		225.4 (68.8, 737.9)				
	dry skin	9	4.0 (2.0, 7.7)	1	7.8 ✗ (1.1, 57.1)	8	9.5 (4.7, 19.3)									
**Respiratory**	pneumothorax	38	48.9 (34.7, 68.9)	2	44.5 ✗ (10.6, 186.6)	4	12.8 (4.7, 34.6)	4	18.7 (6.9, 50.8)	11		104.0 (54.8, 197.5)	2	58.0 ✗ (13.6, 246.7)	13	185.2 (99.6, 344.4)
	pleural effusion	13	9.1 (5.2, 15.8)			5	9.1 (3.7, 22.1)	4	10.6 (3.9, 28.6)	3		14.2 (4.5, 44.9)				
	respiratory failure	12	3.1 (1.7, 5.5)	2	9.4 ✗ (2.2, 39.3)	3	2.0 ✗ (0.6, 6.3)	4	3.9 (1.5, 10.7)	1		10.7 ✗ (1.5, 77.4)			2	4.8 ✗ (1.2, 19.5)
	hypoxia	8	4.4 (2.2, 8.9)	1	9.8 ✗ (1.3, 71.9)	2	2.9 ✗ (0.7, 11.6)								5	27.2 (10.9, 68.2)
	respiratory distress	8	2.6 (1.3, 5.2)			3	2.6 ✗ (0.8, 8.0)			1		2.2 ✗ (0.3, 15.7)			5	16.0 (6.4, 40.1)
**General disorders**	fatigue	23	2.4 (1.6, 3.6)	4	8.0 (2.8, 22.8)	8	2.2 ✗ (1.1, 4.4)	4	1.6 ✗ (0.6, 4.2)	5		3.6 ✗ (1.5, 8.9)	4	10.7 (3.7, 31.1)		
	drug intolerance	18	16.6 (10.3, 26.7)			18	46.2 (28.3, 75.4)									
	mucosal inflammation	15	9.3 (5.6, 15.7)			6	9.8 (4.3, 22.1)	6	14.3 (6.3, 32.6)						3	17.4 (5.4, 55.6)
	asthenia	10	2.4 (1.3, 4.5)	2	8.8 ✗ (2.1, 37.0)	4	2.5 ✗ (0.9, 6.8)	3	2.8 ✗ (0.9, 8.7)	1		1.6 ✗ (0.2, 11.6)				
	general physical health deterioration	7	3.6 (1.7, 7.6)	2	18.9 ✗ (4.5, 79.4)	5	6.8 (2.8, 16.7)									
**Musculoskeletal**	pain in extremity	11	2.9 (1.6, 5.3)	1	4.7 ✗ (0.6, 34.4)	3	2.1 ✗ (0.7, 6.5)	3	3.0 ✗ (0.9, 9.5)	1		1.8 ✗ (0.2, 12.7)	1	6.1 ✗ (0.8, 44.8)	2	4.9 ✗ (1.2, 20.2)
	myalgia	6	3.1 (1.4, 7.0)	1	9.2 ✗ (1.3, 67.7)	1	1.3 ✗ (0.2, 9.6)	2	3.9 ✗ (0.9, 16.0)	1		3.5 ✗ (0.5, 25.1)			1	4.8 ✗ (0.7, 34.4)
	bone pain	5	9.0 (3.7, 21.8)			4	19.0 (7.0, 51.5)								1	16.4 ✗ (2.3, 118.7)
	musculoskeletal pain	5	13.7 (5.6, 33.4)	1	48.4 ✗ (6.6, 356.7)								1	62.6 ✗ (8.4, 464.9)	3	78.5 (24.4, 252.6)
**Gastrointestinal**	diarrhoea	36	3.9 (2.8, 5.5)	4	8.2 (2.9, 23.3)	14	4.0 (2.3, 6.9)	8	3.3 (1.6, 6.7)	8		6.1 (2.9, 12.7)	1	2.4 ✗ (0.3, 17.6)	1	1.0 ✗ (0.1, 6.9)
	stomatitis	11	8.7 (4.8, 15.9)	2	28.7 ✗ (6.8, 120.3)	5	10.4 (4.3, 25.3)	1	2.9 ✗ (0.4, 21.1)	1		5.2 ✗ (0.7, 37.7)			2	14.6 ✗ (3.6, 59.8)
	oesophagitis	5	16.6 (6.8, 40.6)					1	12.7 ✗ (1.8, 91.5)	1		22.2 ✗ (3.1, 160.2)			3	95.2 (29.5, 306.9)
**Metabolism**	decreased appetite	18	2.7 (1.7, 4.4)	2	6.5 ✗ (1.6, 27.4)	9	4.3 (2.2, 8.5)	2	1.4 ✗ (0.3, 5.5)	2		2.4 ✗ (0.6, 9.9)	2	8.5 ✗ (2.0, 36.2)		
	dehydration	11	3.7 (2.1, 6.8)	1	6.0 ✗ (0.8, 44.0)	1	0.9 ✗ (0.1, 6.3)	2	2.6 ✗ (0.6, 10.4)	2		4.6 ✗ (1.1, 18.7)	1	7.8 ✗ (1.0, 57.4)	3	9.6 (3.0, 30.8)
	hypophosphataemia	5	15.7 (6.4, 38.4)	1	55.2 ✗ (7.5, 406.7)					1		20.9 ✗ (2.9, 150.8)	1	71.3 ✗ (9.6, 530.2)	2	58.4 ✗ (14.1, 240.7)
**Vascular**	hypertension	20	4.7 (3.0, 7.4)	1	4.1 ✗ (0.6, 29.9)	10	6.3 (3.3, 11.8)	3	2.6 ✗ (0.8, 8.3)	3		4.7 ✗ (1.5, 15.0)	1	5.3 ✗ (0.7, 39.0)	2	4.3 ✗ (1.0, 17.6)
**Infections**	gastroenteritis	5	5.5 (2.3, 13.2)			4	11.6 (4.3, 31.4)								1	10.1 ✗ (1.4, 72.8)
**Nervous system**	paraesthesia	6	3.2 (1.4, 7.2)			6	8.6 (3.8, 19.4)									
**Endocrine**	hypothyroidism	15	21.3 (12.6, 35.9)	4	107.8 (37.6, 308.7)	1	3.5 ✗ (0.5, 25.2)	4	21.1 (7.8, 57.3)	3		28.2 (8.9, 89.7)	1	31.2 ✗ (4.2, 231.1)	2	25.4 ✗ (6.2, 104.5)

SOC, system organ class; PT, preferred term; ROR, reporting odds ratio; CI, confidence interval; To obtain robust results and reduce the false positive signals, signal values were only calculated for complications with at least 3 records. A signal was defined as both χ^2^ > 4 and lower 95% CI > 1. Negative signals were highlighted in white with✗.

#### 3.3.1 Investigations

Increased alanine aminotransferase (ALT) was the most commonly reported PT (ROR 5.0, 95%CI [3.1–8.1], n = 17) for the SOC level, followed by increased aspartate aminotransferase (AST; ROR 3.5, 95%CI [1.9–6.4], n = 11; Table 2). The strongest signal in this step was increased thyroid stimulating hormone (TSH, ROR 23.5, 95%CI [10.3–53.5], n = 6), and cabozantinib contributed most of the cases (ROR 138.8, 95%CI [55.3–348.1], n = 5). Although the increased blood pressure was a risk signal according to the combined result, no single VEGFR-TKI had a risk definition.

#### 3.3.2 Blood and lymphatic system disorders

Blood and lymphatic system disorders (27.27%, 153 of 561) at the SOC level were also prevalent. Among this SOC in Table 2, anemia had the most cases (ROR 6.9, 95%CI [4.4–10.6], n = 21) and bone marrow failure (ROR 12.4, 95%CI [11.1–25.8], n = 23) presented a strong signal, followed by lymphopenia (ROR 11.6, 95%CI [5.5–24.6], n = 7). Sorafenib gave strong signals for all 9 PTs, especially bone marrow failure (ROR 36.2, 95%CI [22.2–59.0], n = 18), pancytopenia (ROR 29.8, 95%CI [18.7–47.4], n = 20) and lymphopenia (ROR 26.6, 95%CI [11.7–60.2], n = 6). Furthermore, lenvatinib had stronger signals for anemia (ROR 15.6, 95%CI [6.2–39.0], n = 5), febrile neutropenia (ROR 24.2, 95%CI [11.9–49.2], n = 9) and neutropenia (ROR 8.3, 95%CI [3.0–22.9], n = 4) than the other VEGFR-TKI.

#### 3.3.3 Skin and subcutaneous tissue disorders

Cases of skin and subcutaneous tissue disorders (26.20%, 147 of 561) were second only to injury, poisoning, and procedural complications. In the SOC level of Skin and subcutaneous tissue disorders, palmar-plantar eythrodysesthesia syndrome (PPES) was the most frequent PT with the highest ROR (340.9, 95%CI [229.2–507.0], n = 41) and sorafenib contributed most of the PPES cases and had the strongest signal (ROR 745.4, 95%CI [484.4–1,146.9], n = 34). In addition, the high RORs were also detected in alopecia (ROR 10.9, 95%CI [7.1–16.8], n = 22) and hair color changes (ROR 143.4, 95%CI [73.3–280.5], n = 11). For hair color changes, both pazopanib (ROR 220.6, 95%CI [86.5–562.7], n = 5) and cabozantinib (ROR 225.4, 95%CI [68.8–737.9], n = 3) presented high-risk signals respectively.

#### 3.3.4 Other SOCs

Moreover, VEGFR-TKI showed a strong signal for pneumothorax (ROR 48.9, 95%CI [34.7–68.9], n = 38; Table 2), particularly for cabozantinib (ROR 104.0, 95%CI [54.8–197.5], n = 11) and lenvatinib (ROR 185.2, 95%CI [99.6–344.4], n = 13). In musculoskeletal disorders, the signal of musculoskeletal pain was also with a high ROR (13.7, 95%CI [5.6–33.4], n = 5), particularly in lenvatinib (ROR 78.5, 95%CI [24.4–252.6], n = 3). And oesophagitis of gastrointestinal disorders (ROR 95.2, 95%CI [29.5–306.9], n = 3) in lenvatinib was higher than in any other VEGFR-TKI. In endocrine disorders, hypothyroidism presented the highest ROR (21.3, 95%CI [12.6–35.9], n = 15), especially for sunitinib (ROR 107.8, 95%CI [37.6–308.7], n = 4).

#### 3.3.5 Subgroup analysis

As shown in Figure 1. Pediatrics in the younger age group (1–11 years) and the older age group (12–17 years) had similar results in SOC level AEs such as respiratory, thoracic, and mediastinal disorders and blood and lymphatic system disorders. However, the younger age group had a higher proportion of blood and lymphatic system diseases (35.05%, 68 of 194) than the older group (21.80%, 80 of 367). In comparison, the older group had a lower proportion of skin and subcutaneous tissue diseases (27.79%, 102 of 367). AEs in specific PT levels are shown in Table 3. PPES had a higher risk signal in both groups (ROR 222.70 and 324.26, respectively). For hypothyroidism, pediatrics in the younger group showed a significant risk signal (ROR 35.65, 95%CI [17.34–73.30], n = 8), whereas the older group had a strong risk signal for pneumothorax (ROR 71.63, 95%CI [47.83–107.27], n = 30).

**TABLE 3:** Subgroup analysis of the top 10 specific risk signals in the younger age group (1–11 years) and older age group (12–17 years) for VEGFR-TKI.

PT	ROR	95% CI	Case reports (%)	
1–11 (n = 194)				
drug intolerance	43.95	26.10–74.01	16	8.25
bone marrow failure	26.19	15.38–44.58	15	7.73
pancytopenia	20.98	12.34–35.66	15	7.73
skin toxicity	252.87	136.88–467.13	14	7.22
lymphadenopathy	24.26	13.75–42.82	13	6.70
ALT increased	9.32	5.19–16.73	12	6.19
pleural effusion	18.87	9.93–35.83	10	5.15
AST increased	6.76	3.33–13.74	8	4.12
hypothyroidism	35.65	17.34–73.30	8	4.12
PPES	222.70	100.64–492.81	8	4.12
**12–17 (n = 367)**				
PPES	324.26	201.59–521.57	33	8.99
pneumothorax	71.63	47.83–107.27	30	8.17
diarrhoea	5.54	3.76–8.16	28	7.63
pyrexia	2.33	1.52–3.59	22	5.99
Rash	2.60	1.67–4.04	21	5.72
anaemia	10.20	6.39–16.28	19	5.18
febrile neutropenia	10.07	6.23–16.26	18	4.90
alopecia	9.65	5.90–15.78	17	4.63
Fatigue	2.08	1.27–3.38	17	4.63
hypertension	4.93	2.88–8.44	14	3.81

## 4 Discussion

Malignancies in pediatrics present a significant challenge to physicians, partly because of the rarity of occurrence and the relative scarcity of data compared with adult tumors. Vincristine, cyclophosphamide, and irinotecan are chemotherapeutic drugs that have emerged as part of therapeutic regimens in various solid tumors in the last 60 years (Tsakatikas et al., 2021). As previous clinical studies described, combing anti-angiogenesis therapy could provide strong synergistic effects (Weiss et al., 2020; Gaspar et al., 2021b; Xu et al., 2021). Gradually, these drugs have become an increasingly common practice in the setting of limited pediatric oncology treatment options. Most anti-angiogenic treatments targeting VEGFR in pediatrics have been investigated up to phase Ⅰ/II study, and at least four of these relevant clinical studies discuss safety in detail (Wetmore et al., 2016; Verschuur et al., 2019; Brose et al., 2021; Geoerger et al., 2021). Of the VEGFR-TKI included in our analysis, only cabozantinib has been approved by the FDA for children over 12 with differentiated thyroid cancer (DTC) (Duke et al., 2022). Therefore, clinicians’ awareness of common and uncommon VEGFR-TKI-related AEs is of great importance to improve the quality of healthcare for pediatrics.

According to the SOC conducted by disproportionality analysis, the proportion of blood and lymphatic system disorders and skin and subcutaneous tissue disorders in children was significantly higher than in the general population, but the gastrointestinal disorders proportion was significantly lower. Except for axitinib, without any risk signal being detected due to its limited case of uses, the safety profiles of each VEGFR-TKI were not similar for the limited data and biased drugs used. Notably, although sunitinib presented a low RSR, its safety in children remained to be further studied, considering only 22 cases with 124 PT reports.

At the SOC level, although ALT and AST increases were universal for AEs among these drugs in the investigations, few severe hepatobiliary disorders were detected compared to the general population. Skin and subcutaneous tissue disorders and blood and lymphatic system disorders were the most common SOCs in pediatrics. As for blood and lymphatic system disorders, anemia presented a high signal in many cases. In clinical practice, anemia is a common but often underestimated and undertreated event. Anemia and its related fatigue are associated with poor prognosis in cancer patients and were shown to be correlated with a 65% overall increase in the risk of mortality (Harper and Littlewood, 2005; Lang et al., 2017). Barni S et al. revealed that TKIs were associated with higher and more significant risk ratios (RR) (Barni et al., 2012). One possible explanation was that blockade of FLT-3 and Kit by TKIs leads to anemia-induced hematopoietic insufficiency (Weisel et al., 2007; Kent et al., 2008; Williams et al., 2013; Lang et al., 2017). Secondly, microvascular thrombotic hemolytic anemia was also found in some studies (Talebi et al., 2012; Haksöyler and Paydaş, 2021). In addition, we also found that bone marrow failure, lymphopenia, pancytopenia, *etc.*, 9 PTs were risk AEs. However, given the high risk of etoposide (ROR 23.21) in combined use and being reported as a primary suspect drug, the relationship between the risk and VEGFR-TKI remains to be clarified.

Our analysis of the single AE found that the PPES, also named hand-foot skin reaction (HFSR), was the most frequent in pediatrics, followed by pneumothorax and diarrhoea, with over 30 case reports.

Among the 50 included PTs, the PPES signal was the strongest. We observed that sorafenib contributed to most of the cases, and among all included VEGFR-TKI, only sorafenib was judged to be associated with the occurrence of PPES; the signal was much higher than other agents. VEGFR was reported to be the primary cause of PPES, which was consistent with the findings of our study (Lacouture et al., 2008; Chanprapaph et al., 2016). In addition, significantly improved clinical benefit was found in this population compared to patients with advanced HCC who did not develop the HFSR (Vincenzi et al., 2010). The mechanism of PPES is not yet clear cause PPES most commonly occurs on palmoplantar surfaces, so in general, PPES is related to the repair of skin damage (Lacouture et al., 2008; Chanprapaph et al., 2016). Some previous studies have proved that its occurrence may result from a combination of multiple factors. First, TKI is cytotoxic to keratinocytes (Yamamoto et al., 2014; Zimmerman et al., 2016), and second, it also inhibits angiogenesis in wound repair (Eming and Krieg, 2006; Apte et al., 2019). Third, inhibiting immune downmodulate after VEGF is inhibited may also lead to one of the factors of PPES(Motz and Coukos, 2013; Apte et al., 2019). Effective wound repair would be hampered by the inhibition of VEGF in downregulating immune responses brought on by these agents, which could result in PPES.

Intriguingly, we discovered that the pneumothorax signal following VEGFR-TKI use in kids was second only to PPES. This phenomenon was generally prevalent in the six included VEGFR-TKI and significantly higher than the signal in the general population (ROR 6.09). The signal strength was also significantly more than that of the same event in adults and not listed on the label. In two retrospective studies and a phase II single-arm multicenter study involving anti-angiogenesis agents used in children and adolescents, the incidence of pneumothorax during treatment ranged from 13.3% to 33% (Interiano et al., 2015; Italiano et al., 2020; Bodea et al., 2022). Subsequent research proved peripheral lung lesions or necrosis of pleural lesions due to the tumor directly involving the pleura after chemotherapy might be the cause of pneumothorax rather than the direct toxicity of chemotherapy drugs (Sabath et al., 2018; Aiba et al., 2021), and solid tumors account for 30% of all pediatric cancers (Spini et al., 2022). Factually, after reviewing the raw data, we found the indications of cases who reported pneumothorax with VEGFR-TKI were all soft tissue sarcoma. Second, the ROR results for the drugs in the combined medications, like ifosfamide, etoposide, everolimus, *etc.*, were much lower when the above method was used to calculate them than the former. The existing literature confirmed that sarcoma usually metastasizes with a stable frequency, most children treated with VEGFR-TKI are already advanced, and lung metastases are more likely (Burnei et al., 2013; Interiano et al., 2015; Murugan et al., 2018; Andión Catalán et al., 2020). Considering the data from FAERS depends on spontaneous reporting, a significant bias would be presented after correlating the presence or not of metastases from sarcoma with the occurrence of pneumothorax. Thus, in conjunction with the previous article, the incidence of pneumothorax is associated with the clinical benefit of using these drugs, but due to its high risk, one should be alert and preventative against the occurrence of this AE during therapy with VEGFR-TKI.

We found that cabozantinib carries a higher risk signal of musculoskeletal pain than other drugs and that lenvatinib also carries a greater risk of oesophagitis. But because these two AEs were only reported in a small number of children, the final ROR seemed too high. More research needs to be done to determine if two AEs are linked to using VEGFR-TKI. In addition, we also noticed that VEGFR-TKI, especially sunitinib, pazopanib, and cabozantinib, presented a significant signal of hypothyroidism. The possible mechanisms of TKI-induced hypothyroidism include the degeneration of the capillaries in the thyroid; the TKI-induced apoptosis of the thyroid follicular cells; and altered thyroid hormone metabolism (Liao et al., 2021; Basolo et al., 2022). These results showed that TKI-induced hypothyroidism was more related to thyroid atrophy caused by the first two situations. Therefore, because of the effect on child growth, thyroid function needs to be closely monitored when using VEGFR-TKI, then reduced or stopped according to the situation above.

However, due to its characteristics, FAERS data only represents a portion of the healthcare population. The trial sponsor, affected participant, and general practitioner may report this data because it was spontaneously reported. The information on disease severity or outcomes is lacking, and the data may contain duplicate, incomplete, inaccurate, and omitted reports. Second, we utilized the ROR method in the analysis. Although the ROR method is simple and easy to understand, the results are highly susceptible to individual values. The statistic fluctuates greatly if the cell frequency is small. Thus, when signal detecting according to the standard, we focused on AEs with many cases to avoid false positives. Finally, Due to the particularity of children, VEGFR-TKIs are usually combined with conventional radiotherapy and chemotherapy for advanced-stage tumors for children in clinical practice. They are rarely used as a monotherapy. Therefore, the sensitivity analysis of these concomitant drugs’ effect on this population could not be performed.

Overall, our study showed that the risk profiles of VEGFR-TKI vary. By analyzing the comprehensive characterization of these drugs based on the FAERS, new, severe, or unexpected AE signals can be identified. Although using the FAERS database has limitations, the comparative exploration of VEGFR-TKI and background factors through disproportionality analysis can partially avoid the influence of confounding factors in cancer patients (Uetake et al., 2018). These results can provide evidence for future clinical research and enable clinicians to choose the optimal therapies in clinical practice.

## 5 Conclusion

The present study explored the safety profile of VEGFR-TKI in pediatrics using the FAERS database. Multiple skin and subcutaneous tissue disorders, as well as blood and lymphatic system disorders, were common VEGFR-TKI-related AEs in SOC. No serious hepatobiliary AEs were detected. For the specific AEs, PPES and pneumothorax were VEGFR-TKI-related AEs that presented significantly higher signals than those in the general population. Future observational studies, population cohorts, and clinical trials are required to validate the AEs of pediatric VEGFR-TKI used off-label.

## Data Availability

The original contributions presented in the study are included in the article/Supplementary Material, further inquiries can be directed to the corresponding author.
