# Supplementary material for: Safety profile of vascular endothelial growth factor receptor tyrosine-kinase inhibitors in pediatrics: a pharmacovigilance disproportionality analysis
Source: Front Pharmacol. 2023 Jun 12;14:1160117. doi: 10.3389/fphar.2023.1160117 (PMC10291139; doi:10.3389/fphar.2023.1160117)
Supplement: Supplementary file 1 [file DataSheet3.docx]

***Supplementary Material***

Safety Profile of Vascular Endothelial Growth Factor Receptor Tyrosine-kinase Inhibitors in Pediatrics: A Pharmacovigilance Disproportionality Analysis

Yifei Xue, Shuo Feng, Guangyao Li, Chao Zhang*

*** Correspondence:** Chao Zhang: laural.zhang@yahoo.com

# Supplementary Table:

**Supplementary Table 1:** The 2×2 crosstab of ROR

|  | **Drug of interest** | **Other drugs** |
| --- | --- | --- |
| **AEs of interest** | DE | dE |
| **Other AEs** | De | de |

ROR, reporting odds ratio; DE, the number of interest drug reports for suspect AE; dE, the number of other drugs reports for suspect AE; De, the number of interest drug reports for other AE; de, the number of other drugs reports for other AE.

**Supplementary Table 2:** Front four indications based on pediatric cases of using VEGFR-TKIs

| **Drugs** | **Indications** | **Cases n, (%)** | |
| --- | --- | --- | --- |
| VEGFR-TKIs | Osteosarcoma | 85 | (15.15) |
|  | Acute myeloid leukaemia | 46 | (8.20) |
|  | Hepatocellular carcinoma | 40 | (5.20) |
|  | Ewing's sarcoma | 21 | (3.74) |
|  | **total** | **561** | **(100)** |
| Sorafenib | Acute myeloid leukaemia | 41 | (7.31) |
|  | Hepatocellular carcinoma | 25 | (4.46) |
|  | Desmoid tumour | 12 | (2.14) |
|  | Osteosarcoma | 11 | (1.96) |
|  | **total** | **209** | **(37.25)** |
| Sunitinib | Gastrointestinal stromal tumour | 3 | (0.53) |
|  | Renal cell carcinoma | 3 | (0.53) |
|  | Desmoplastic small round cell tumour | 2 | (0.36) |
|  | Thyroid cancer | 2 | (0.36) |
|  | **total** | **31** | **(5.53)** |
| Pazopanib | Bone cancer | 11 | (1.96) |
|  | Osteosarcoma | 8 | (1.43) |
|  | Rhabdomyosarcoma | 8 | (1.43) |
|  | Desmoid tumour | 5 | (0.89) |
|  | **total** | **143** | **(25.49)** |
| Cabozantinib | Osteosarcoma | 17 | (3.03) |
|  | Bone cancer | 10 | (1.78) |
|  | Ewing's sarcoma | 10 | (1.78) |
|  | Renal cancer | 8 | (1.43) |
|  | **total** | **84** | **(14.97)** |
| Lenvatinib | Osteosarcoma | 28 | (4.99) |
|  | Hepatocellular carcinoma | 3 | (0.53) |
|  | Nephroblastoma | 3 | (0.53) |
|  | Ewing's sarcoma | 3 | (0.53) |
|  | **total** | **60** | **(10.70)** |
| Regorafenib | Osteosarcoma | 12 | (2.14) |
|  | Bone cancer | 2 | (0.36) |
|  | Gastrointestinal stromal tumour | 1 | (0.18) |
|  | Adenocarcinoma of colon | 1 | (0.18) |
|  | **total** | **24** | **(4.28)** |
| Axitinib | Osteosarcoma | 2 | (0.36) |
|  | Alveolar soft part sarcoma metastatic | 2 | (0.36) |
|  | Adrenal carcinoma | 1 | (0.18) |
|  | Renal cancer | 1 | (0.18) |
|  | **total** | **10** | **(1.78)** |

**Supplementary Table 3:** Distribution of potential risk PTs attributed to VEGFR-TKIs in the SOC category of all populations

| **SOC** | **General Population** | | | **Pediatrics** | | |
| --- | --- | --- | --- | --- | --- | --- |
|  | **PTs (n)** | **Case n, (%)** | | **PTs (n)** | **Case n, (%)** | |
| Neoplasms benign, malignant and unspecified | 161 | 10409 | (19.30) | 12 | 121 | (21.57) |
| Gastrointestinal disorders | 139 | 18249 | (33.84) | 5 | 60 | (10.70) |
| Investigations | 113 | 12020 | (22.29) | 14 | 101 | (18.00) |
| Skin and subcutaneous tissue disorders | 55 | 8893 | (16.49) | 11 | 147 | (26.20) |
| Infections and infestations | 51 | 773 | (1.43) | 9 | 32 | (5.70) |
| Respiratory, thoracic, and mediastinal disorders | 38 | 3096 | (5.74) | 6 | 82 | (14.62) |
| Hepatobiliary disorders | 38 | 4023 | (7.46) |  |  |  |
| Vascular disorders | 32 | 4805 | (8.91) | 3 | 26 | (4.63) |
| General disorders and administration site conditions ^a^ | 30 | 20668 | (38.33) | 6 | 153 | (27.27) |
| Metabolism and nutrition disorders | 27 | 6448 | (11.96) | 4 | 38 | (6.77) |
| Nervous system disorders | 26 | 2400 | (4.45) | 1 | 6 | (1.07) |
| Injury, poisoning, and procedural complications | 28 | 404 | (0.75) | 5 | 283 (4) ^c^ | (0.71) |
| Reproductive system and breast disorders | 23 | 231 | (0.43) |  |  |  |
| Endocrine disorders | 23 | 1512 | (2.80) | 1 | 15 | (2.67) |
| Surgical and medical procedures | 20 | 244 | (0.45) | 2 | 11 | (1.96) |
| Blood and lymphatic system disorders | 18 | 1389 | (2.58) | 10 | 153 | (27.27) |
| Renal and urinary disorders | 20 | 1718 | (3.19) | 2 | 10 | (1.78) |
| Musculoskeletal and connective tissue disorders | 12 | 317 | (0.59) | 4 | 27 | (4.81) |
| Cardiac disorders | 10 | 340 | (0.63) | 1 | 5 | (0.89) |
| Eye disorders | 5 | 51 | (0.09) |  |  |  |
| Psychiatric disorders | 2 | 230 | (0.43) |  |  |  |
| Immune system disorders | 1 | 6 | (0.01) | 3 | 8 | (1.43) |
| **Total** ^b^ | **872** | **53921** | **(100)** | **99** | **561** | **(100)** |

SOC, system organ class; PT, preferred terms; a, death and disease progression were not included in the general disorders and administration site conditions; b, the total part is recorded as the number of PTs and cases obtained after data mining, not the sum of the cases in this table; c, the results in brackets related to off-label use were excluded in case statistics.

**Supplementary Table 4:** Disproportionality analysis results of each agent. Risk PTs are the report PTs which are detected as VEGFR-TKI related AEs

| **VEGFR-TKI** | **General Populations** | | | **Pediatrics** | | |  |
| --- | --- | --- | --- | --- | --- | --- | --- |
|  | **Risk PTs**  **(n)** | **All PTs**  **(n)** | **Proportion**  **(%)** | **Risk PTs**  **(n)** | **All PTs**  **(n)** | **Proportion**  **(%)** | ***p*** |
| Sunitinib | 476 | 2894 | 16.45 | 11 | 125 | 8.80 | 0.03* |
| Sorafenib | 562 | 2967 | 18.94 | 69 | 303 | 22.77 | 0.10 |
| Regorafenib | 365 | 1787 | 20.43 | 10 | 73 | 13.70 | 0.18 |
| Pazopanib | 327 | 2336 | 14.00 | 20 | 198 | 10.10 | 0.13 |
| Cabozantinib | 312 | 1970 | 15.84 | 22 | 142 | 15.49 | 0.91 |
| Lenvatinib | 284 | 1817 | 15.63 | 23 | 95 | 24.21 | 0.02* |
| Axitinib | 177 | 1917 | 9.23 | 0 | 24 | 0 |  |

*, the Chi-square test indicates a statistically significant difference (p<0.05) between the general population and children
